# A hierarchical machine learning framework for the analysis of large scale animal movement data

**DOI:** 10.1186/s40462-021-00242-0

**Published:** 2021-02-18

**Authors:** Colin J. Torney, Juan M. Morales, Dirk Husmeier

**Affiliations:** 1 School of Mathematics and Statistics, University of Glasgow, Glasgow, G12 8SQ UK; 2 Grupo de Ecología Cuantitativa, INIBIOMA, Universidad Nacional del Comahue, CONICET, Düsternbrooker Weg 20, Bariloche, S4140 Argentina

**Keywords:** Animal movement, Machine learning, Large-scale data

## Abstract

**Background:**

In recent years the field of movement ecology has been revolutionized by our ability to collect high-accuracy, fine scale telemetry data from individual animals and groups. This growth in our data collection capacity has led to the development of statistical techniques that integrate telemetry data with random walk models to infer key parameters of the movement dynamics. While much progress has been made in the use of these models, several challenges remain. Notably robust and scalable methods are required for quantifying parameter uncertainty, coping with intermittent location fixes, and analysing the very large volumes of data being generated.

**Methods:**

In this work we implement a novel approach to movement modelling through the use of multilevel Gaussian processes. The hierarchical structure of the method enables the inference of continuous latent behavioural states underlying movement processes. For efficient inference on large data sets, we approximate the full likelihood using trajectory segmentation and sample from posterior distributions using gradient-based Markov chain Monte Carlo methods.

**Results:**

While formally equivalent to many continuous-time movement models, our Gaussian process approach provides flexible, powerful models that can detect multiscale patterns and trends in movement trajectory data. We illustrate a further advantage to our approach in that inference can be performed using highly efficient, GPU-accelerated machine learning libraries.

**Conclusions:**

Multilevel Gaussian process models offer efficient inference for large-volume movement data sets, along with the fitting of complex flexible models. Applications of this approach include inferring the mean location of a migration route and quantifying significant changes, detecting diurnal activity patterns, or identifying the onset of directed persistent movements.

**Supplementary Information:**

The online version contains supplementary material available at (10.1186/s40462-021-00242-0).

## Introduction

Animal movement is a fundamental ecological process that influences the dynamics of ecosystems across multiple spatiotemporal scales. Movement determines individual fecundity and survival, affects population dynamics and persistence, and alters the trophic interactions between species [1, 2]. Altered animal movement patterns are also an important indicator of the impacts of climate change and the effects of increased anthropogenic land-use [3].

Over recent years there has been a rapid advance in our ability to collect data on the movement behaviour of many animal species [4, 5] and this has led to the development of sophisticated statistical methods to analyse these data [6]. Statistical methods usually model animal movement as some form of random walk which can include directional persistence (correlated random walk), or different forms of biases [7, 8], an approach which enables inference of model structure and parameters.

Broadly speaking, random walk models may be divided into continuous-time and discrete-time models. Discrete-time models are intuitive and readily interpretable but results are often sensitive to the choice of discretization step. Continuous-time movement models are able to cope with irregular sampling intervals and likely better represent the dynamic decision processes of most organisms. Furthermore, continuous time models usually make it easier to deal with measurement error [9].

Both approaches lead to significant computational challenges when fitting to data. Remote telemetry devices generate large, high-frequency data sets and statistical methods must be scalable and able to process and analyze these data sets on practical time scales.

Within the field of machine learning there has traditionally been a focus on developing methods that can be applied at scale. While historically this focus has often come at the cost of formal uncertainty quantification and interpretability, modern machine learning methods combine scalable algorithms with probabilistic learning from data [10]. A popular machine learning technique for the analysis of time series data is Gaussian process regression [11] (also known as kriging within the spatial statistics community).

Gaussian processes are powerful tools for investigating structure and patterns in time series data [12, 13] and have been recently applied to animal movement [14]. In standard Gaussian process (GP) regression we begin with a series of *n* input-ouput datapoints (*t*_*i*_,*x*_*i*_) for $i = 1,\ \dots n$ and assume a relationship between input and output of the form,

1 $$ x(t) = f(t)+ \epsilon   $$

where $\epsilon \sim \mathcal {N}(0,\sigma ^{2}_{m})$ is a white additive noise term, normally associated with measurement error, and *f*(*t*) is a latent (unobserved) function.

The aim is then to infer a posterior distribution over possible functions *f*(*t*) given the observed data. This is achieved by placing a Gaussian process prior on the latent function

2 $$ f(t) \sim \mathcal{GP}\left(m(t), K(t,t') \right)   $$

where *m*(*t*) is a mean function and *K*(*t*,*t*^′^) is a covariance function (or kernel). By assuming that the latent function is a realization of a Gaussian process we can make use of Bayes’ rule and Gaussian identities to calculate the posterior distribution over functions once data has been observed [11].

The choice of covariance function *K*(*t*,*t*^′^) and its parameters plays a key role in the performance of Gaussian process regression. Learning from data for GP regression involves maximizing the marginal likelihood of the data over the covariance kernel hyperparameters and observation noise, approximating the posterior distribution with variational based methods, or sampling from the posterior with Markov chain Monte Carlo.

Placing a Gaussian process prior on the latent function *f*(*t*) is equivalent to assuming the observations are generated from a process that can be written as a linear stochastic differential equation (SDE); it can be shown that the choice of covariance kernel specifies the form of the equivalent SDE for the generating process [15]. In the context of animal movement this means that random walk movement models such as those based on Brownian motion or the Ornstein-Uhlenbeck process [8] are formally equivalent to Gaussian processes [16]. For example, for an Ornstein-Uhlenbeck process, the appropriate covariance function is the Matérn 12 function (commonly known as the exponential covariance function),

3 $$ K_{OU}(t, t')=\sigma_{k} \exp{\left(-\frac{|t-t'|}{L}\right)}   $$

where *L* is the reciprocal of the mean reversion parameter (see [17] Section 6.4 for a discussion of the effect of *L*,*σ*_*k*_ on the resulting function *f*(*t*)).

Recent efforts in animal movement modeling have focused on dynamic models in which the parameters of the process change over time. The dominant approach in this context is to assume that the observed movements of animals are driven by discrete behavioural states (eg. foraging, migrating, resting) that the animal switches between at different times [18–20]. For discrete-time models, the usual approach belong to the class of hidden Markov models (HMMs). An HMM is a time series model that comprises two components, an observable series and an underlying, non-observable state sequence. The observed data are taken to be conditionally independent given the states and are generated by so-called state-dependent distributions. The state sequence is modeled as a Markov process usually assumed to be of first order, which means that the probability of state occurrences at time t+1 depends only on which state the chain is in at time t [21, 22].

An equivalent discrete-state process can be modelled using Gaussian process regression either via the introduction of change points [23] or by directly employing a hidden Markov model [24]. However, in many cases we expect that changes in movement would be more gradual, for example as individuals respond to their environment or internal condition as they move around [25–27]. In this work we employ a continuous state approach through the use of a non-stationary Gaussian process [28], where the parameters of the covariance kernel (sensu movement model) change continuously over time.

We apply the non-stationary Gaussian process approach to the analysis of simulated and real animal movement data and show that this allows us to infer continuous latent behavioural states and encode multiscale periodic models that can be rigourously and efficiently fit to large data sets.

## Methods

Our regression model follows the 2-dimensional version of Eqs. 1 and 2, where we model each output of the multivariate Gaussian process as two univariate GPs. This can be generalised to multivariate GPs where the correlation between outputs is explicitly modelled, [29, 30] however for animal movement data the difference is likely to be negligible.

We further replace the stationary kernel defined by Eq. 3 with a non-stationary version of the Matérn 12 covariance function [31],

4 $$ \begin{aligned} K_{NS}(t, t') &=\sqrt{\frac{2\sigma^{2}(t)\sigma^{2}(t')L(t)L(t')}{L(t)^{2}+L(t')^{2}}}\\ &\quad\times \exp{\left(-\sqrt{\frac{2(t-t')^{2}}{L(t)^{2}+L(t')^{2}}}\right)} \end{aligned}  $$

so that the parameters of the kernel vary as a function of *t*.

Following, [28] we model the latent lengthscale and kernel amplitude using lower level Gaussian processes so that,

5 $$ \tilde{\sigma}(t) \sim \mathcal{GP}\left(\mu_{\sigma}, K_{\sigma}(t,t') \right) \\ \tilde{L}(t) \sim \mathcal{GP}\left(\mu_{L}, K_{L}(t,t') \right)  $$

where $\tilde {\sigma }, \tilde {L}$ undergo an exponential transformation to obtain positive values for *σ* and *L*.

In relation to previous approaches that employ an Ornstein-Uhlenbeck (OU) process to model position or velocity [32, 33], conceptually our framework replaces the standard definition of such a process,

6 $$ d\mathbf{x} = -\nu\, (\mathbf{x}-\mathbf{m}) dt + \eta \ d\mathbf{W}_{t}  $$

where *ν* is the mean reversion rate, **m** is the mean value, *η* is the noise amplitude, and **W**_*t*_ is a Wiener process, with an OU process with time-varying parameters,

7 $$ d\mathbf{x} = -\nu(t)\, \left(\mathbf{x}- \mathbf{m}(t)\right) dt + \eta(t) \ d\mathbf{W}_{t}   $$

where the parameters of the covariance function above can be related to the parameters *ν*(*t*),*η*(*t*) as in the stationary case [34],

8 $$ \text{\L}(t) \approx \frac{1}{\nu(t)}, \quad \sigma(t)^{2} \approx \frac{\eta(t)^{2}}{2\nu(t)}.  $$

We further model the mean function of the process as

9 $$ \mathbf{m}(t) \sim \mathcal{GP}\left(0, K_{m}(t,t') \right)  $$

and within our framework, we are free to assume any, all, or none of the parameters are dynamically varying, where in the latter case we recover a standard OU process.

The covariance kernels of the lower level GPs control the smoothness and structure of the latent functions. Appropriate choice of these covariance kernels allows us to encode structure into the models, such as periodicity, and combine kernels together [35], for example to model a periodic movement pattern (representing an annual migration) with a long term trend (representing a shifting migration route).

Once the kernels of the lower level GPs have been defined the unnormalised posterior probability of the trajectory data can be calculated as,

10 $$\begin{array}{@{}rcl@{}} \mathcal{N}\left(\boldsymbol{x} \mid \mathbf{m}, K_{NS} + \sigma^{2}_{m}{I}\right) \mathcal{N}\left(\mathbf{m} \mid \, 0, K_{m} \right) \times \\ \mathcal{N}\left(\tilde{L} \mid \mu_{L}, K_{L} \right) \mathcal{N}\left(\tilde{\sigma} \mid \mu_{\sigma}, K_{\sigma}\right) P(\boldsymbol{\theta}) \end{array} $$

where ***x*** is the matrix of data points, *σ*_*m*_ is the measurement uncertainty, *I* is the identity matrix, ***θ*** is a vector containing the hyperparameters of the lower level Gaussian processes contained in the functions **m**,*σ* and *L*, and *P*(***θ***) is the prior probability over the hyperparameters.

To implement the model we use TensorFlow Probability [36, 37], a machine learning library designed for probabilistic modeling on computational hardware such as GPUs or TPUs. For sampling from the posterior distribution of latent functions and parameters we use an adaptive version of the Metropolis-adjusted Langevin algorithm (MALA) [38], a Markov chain Monte Carlo (MCMC) algorithm that makes use of the gradient of the target probability density function when generating proposals and hence is a more efficient sampler.

As we are interested in learning about the latent function values, i.e. the parameters of the movement process, we determine the latent functions at *m* support points where *m*≪*n* and the locations of the *m* points are chosen to be evenly distributed across the domain of the latent function with the number of points chosen so that the distance between points is less than the correlation lengthscale of the latent function. To improve computational efficiency and avoid the *O*(*n*^3^) scaling with data size typically associated with estimation of GP hyperparameters [11] we firstly make use of the fact that movement data consists of trajectories of individual animals that may be considered conditionally independent given the latent movement parameters. This means our method is at most *O*(*k*^3^) where *k* is the number of data points in the largest individual trajectory.

For high-frequency, long term data sets we further segment individual trajectories into smaller sections of length *j* data points and use a series of local Gaussian processes to approximate the full trajectory. This approach, also known as a mixture of Gaussian process experts [39], gives an accurate approximation provided the domain of the local GP is large compared to the lengthscale of the process being modelled and the data is relatively uniform [40]. We therefore approximate a single long trajectory of length *k* as a sequence of separate smaller trajectories of length *j*, and as a result the computational time for inference is greatly reduced. For example, given a trajectory consisting of hourly GPS fixes over two years, we would have 17280 data points and inference would proceed in *O*(17280^3^) time. Instead, we divide the trajectory into 3 month segments which are conditionally independent given the latent movement parameters and inference then proceeds in *O*(8×(2160^3^)) time.

To evaluate our approach we firstly generate synthetic trajectory data with dynamic, non-stationary movement parameters. We then apply our inference framework and compare inferred values with our known parameters. We next apply the methodology to a real dataset concerning the movement dynamics of free-roaming sheep in Patagonia.

## Results for simulated movement data

Through the use of synthetic data we assess two potential scenarios where our methods will be applicable. We firstly detect daily activity patterns in a simulated individual organism, we then infer changes to a seasonal migration route where an annual movement pattern is shifting over a longer timescale.

### Periodic activity patterns

**Fig. 1:**
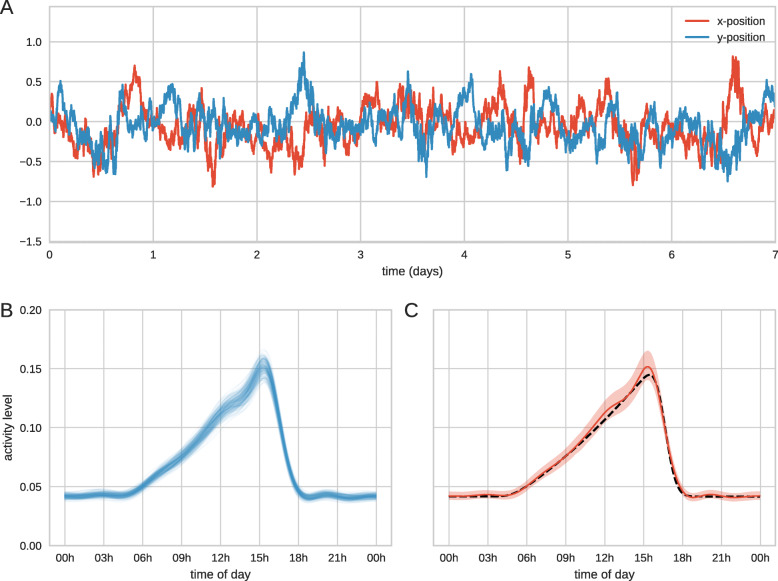
Detection of periodic activity pattern. (**a**) Coordinates of the simulated individual. The amplitude of the random movement increases for the same period each day. (**b**) Samples from the posterior. Using Markov chain Monte Carlo (MCMC) sampling we can sample from the posterior distribution over functions, hence we can infer the latent activity levels as a function of time of day. (**c**) Once we have taken sufficient samples we can calculate the posterior mean (red line) and 95% credible interval (shaded region). The true latent function is shown as a black dashed line

A random walker is simulated as a 2-dimensional Ornstein-Uhlenbeck process with a time-varying amplitude parameter which models an animal moving around a home-range with different activity levels (see Appendix for details). In this simulation we model an individual that has an increasing period of high-activity (e.g. foraging) beginning around 5am and reducing more sharply late afternoon. This gives rise to the synthetic data set shown in Fig. 1a. From this observed data the challenge is to infer the underlying, time-varying movement parameters.

As we are investigating a daily activity pattern, we will encode this into the covariance kernel of the latent process for the OU amplitude. To do so, we will implement a periodic kernel [41] for the amplitude kernel defined as

11 $$ K_{\sigma}(t, t') = a\exp{\left(\frac{-2\sin^{2}(\pi |t - t'| / P)}{l^{2}}\right)}  $$

where *P* defines the period which we will fix to be 24 hours. The parameters *l* and *a* are the length scale and amplitude of the lower level Gaussian process that control the persistence of motion and ranging area, and are inferred along with the latent function values.

All parameters and latent functions are sampled from using the MALA sampler. We therefore infer the three hyperparameters associated with the periodic amplitude, *μ*_*σ*_,*a*,*l*, the constant lengthscale *μ*_*L*_, and 40 latent function values at the support points.

We took 40000 steps that are thinned to give 2000 samples, following a burn-in period of 5000 steps and run 4 independent chains. Potential scale reduction factors (PSRF) [42] were calculated to assess convergence and mixing with the maximum value over all parameters calculated as 1.08. Results are shown in Fig. 1. Here a single sample represents a sample from a distribution over functions, as well as the parameters of the model. Hence we infer the functional form of the amplitude parameter throughout the day. As we have simulated the random walk using an Ornstein-Uhlenbeck process we are able to accurately recover the true latent function (Fig. 1c).

### Altered migration routes

We next simulate a longer term movement process in which a single individual follows a seasonal migration between breeding grounds and a wintering area [43]. We simulate a migration of this type by employing an oscillating mean location with an annual frequency. We also include a slowly shifting Northern range and seek to detect the annual movement pattern and the long term trend. (Full details of the synthetic data generation can be found in the Appendix and an example trajectory is shown in Fig. S1.)

To infer the properties of the movement process we combine periodic and squared exponential kernels to create a periodic kernel that can slowly vary over time [35],

12 $$ \begin{aligned} K_{m}(t, t') &= a \exp{\left(-\frac{(t-t')^{2}}{2\lambda^{2}} \right)}\\ &\quad\exp{\left(\frac{-2\sin^{2}(\pi |t - t'|/P)}{l^{2}}\right)}. \end{aligned}  $$

We set the period to be 1 year and the lengthscale of the squared exponential to be 5 years. In principle these parameters could be inferred from the data as well, however we may expect a strong and reliable prior for seasonal drivers of movement patterns and the timescale over which longer term changes occur.

**Fig. 2:**
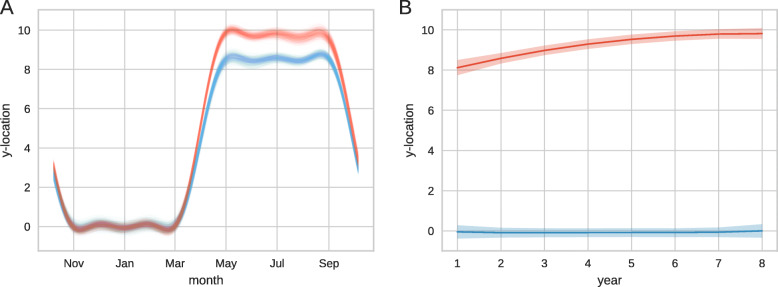
Detection of shifting migration patterns. (**a**) Samples from the posterior distribution of the mean migration route. Samples are shown for the average North-South location at each point during the year for year 1 (blue) and year 7 (red). (**b**) Average location of the summer (red) and winter (blue) areas along with 95% credible intervals. The true value increases from 8 to 10 and is shown in Fig. S1

For inference, we ran 4 independent chains of 80000 steps each then discarded a burn-in period of 40000 steps. The 40000 steps following burn-in are thinned to leave 2000 samples from the posterior (maximum PSRF 1.17). Results from the sampler are shown in Fig. 2. As before we can draw samples of the underlying latent function, which in this example describes the average migration route. We may also estimate the locations of wintering and breeding grounds across years and formally quantify the uncertainty in these estimates (Fig. 2b).

## Case study: free-roaming sheep in Patagonia

Finally, we apply our methods to empirical data collected from GPS collars attached to 27 sheep ranging freely in Patagonia. Sheep movement data was collected at the Pilcaniyeu experimental range station from the Argentine National Institute for Agropecuary Technology (70^∘^ 35 ^′^21^″^W, 41^∘^ 01 ^′^42^″^S) located in the western district of the Patagonian steppe. The 27 Merino ewes were equipped with GPS collars (CatLog-B, Perthold Engineering, www.perthold.de; USA) programmed to register locations every 10 minutes over a 2-month period and allowed to freely roam in a paddock of 700 hectares, resulting in 225,953 data points. Full trajectories were split into segments of 1000 data points (approximately 7 days) and we calculated the velocity of individual sheep at each time point using a finite difference of successive position measurements.

We assume all individuals adopt similar behaviours and use our framework to investigate the daily behavioural patterns of the sheep. We use an OU process to model the velocity of individual sheep with varying amplitude and lengthscale that follows a 24-hour period. Hence, Eq. 7 now models velocity and not location, and the covariance kernel of the lower level Gaussian processes is given by,

13 $$ K_{\sigma}(t, t') = \sigma_{\sigma}\exp{\left(\frac{-2\sin^{2}(\pi |t - t'| / P)}{l_{\sigma}^{2}}\right)}  $$

14 $$ K_{L}(t, t') = \sigma_{L}\exp{\left(\frac{-2\sin^{2}(\pi |t - t'| / P)}{l_{L}^{2}}\right)}  $$

where *P*=24. To determine the kernel hyperparameters of the latent GPs, we minimize the negative log-likelihood of the model using the Adam optimizer [44] on a subset of the data. We then fix these hyperparameters to the optimal values and sample from the posterior distribution of the latent functions and the measurement error using the adaptive MALA algorithm. Note, it is possible to sample from all parameters of the model, including the kernel hyperparameters but in our tests this resulted in slow mixing of the chains and meant convergence was not achieved within reasonable timescales given the size of our dataset.

We ran chains of 25000 steps each. The first 20000 steps were used as burn-in during which time the algorithm adapted the step size and proposal distribution. Following burn-in the remaining 5000 steps were thinned and every tenth sample retained. Each chain took approximately 14 hours to run on a Tesla V100 GPU. To check for convergence we calculated potential scale reduction factors and report effective sample sizes (see Figs. S2 and S3).

**Fig. 3:**
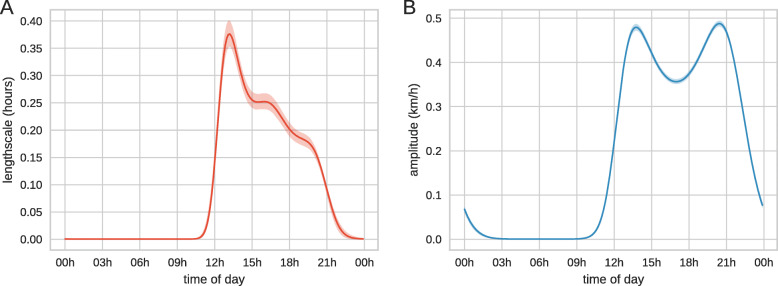
Daily activity patterns of free-roaming sheep. Posterior mean and 95% credible intervals (shaded region) are shown. (**a**) Inferred values for the correlation length of the velocity of all individuals. (**b**) Inferred values for the amplitude of the velocity for all individuals

Inferred latent movement parameters are shown in Fig. 3. These results reveal persistent, high-amplitude movements occur between noon and 11pm, with two clear activity peaks likely representing transit to and from foraging grounds. We also infer the measurement error due to the devices and the posterior distribution of the velocity estimate error is shown in Fig. S4. When inferred velocity is low any movements are attributed to measurement error. This results in higher uncertainty in these periods although this is only visible when examining the untransformed latent functions as shown in Fig. S5.

Our framework is able to characterise a clear activity pattern in the flock and reveals the daily dynamics of feeding and encampment. To validate the model we perform posterior predictive checks by comparing the distributions of steps and turns from the discretized empirical data and simulated data created using the inferred time-varying velocity model.

Results comparing the step-and-turn characteristics of simulated trajectories with real trajectories can be found in Fig. S6. These results show our model accurately captures the aggregate distribution of step sizes but there is lower variability and an excess of reversals in the empirical data. The lack of reversals is likely due to a combination of the momentum that is inherent in continuous time velocity models [9], along with the effects of environmental features and site fidelity in the sheep, factors that are absent in our model.

## Discussion

In recent years, the field of movement ecology has been transformed by the availability of accurate, high-frequency telemetry data. Despite this availability of data, we are still left with the significant challenge of inferring the dynamics of large scale ecological processes from small numbers of collared individuals.

A major component of this challenge arises because animal movement is inherently a complex, multiscale process [45], that is driven by periodic drivers [46], environmental cues [47], social interactions [48, 49] and individual memory [50, 51]. Aside from this complexity, statistical difficulties arise due to the autocorrelated nature of movement data. Movement models account for autocorrelation through the use of random walk models [33, 52, 53] and much effort has been made to develop methods to fit these models to movement data [6].

Learning from large scale datasets is the primary goal of many machine learning methods and the rapid growth of ecological data has led to machine learning being increasingly viewed as an essential component of the ecologist’s toolbox [54, 55]. Traditional applications of machine learning have focused on tasks where formal uncertainty quantification is not a main aim, such as computer vision tasks [56] or the classification of accelerometer data [57]. However, probabilistic machine learning methods [58], such as Gaussian processes, offer a way to analyse data, infer parameters, and quantify uncertainty within a non-parametric Bayesian framework.

There are several advantages to applying these techniques to animal movement modelling. Practically speaking, machine learning libraries are scalable to large datasets, well-supported by a community of researchers, and run on modern HPC hardware. Deep learning libraries such as TensorFlow [37] use automatic differentiation that makes calculating the gradients of the likelihood with respect to model parameters straightforward. This facilitates optimisation of parameters to find maximum likelihood estimates, as well as more efficient Monte Carlo sampling algorithms, such as Hamiltonian Monte Carlo [59] sampling or the Metropolis-adjusted Langevin algorithm [60] used here. Gaussian process models can also be implemented in many other statistical software packages, such as STAN or JAGS, meaning it would be relatively straightforward to implement the method we describe here using generic statistical libraries.

In our approach, we model both the movement process and the dynamic parameters of the movement as Gaussian processes. This can be considered a continuous state version of a hidden Markov movement model [18, 21] with the potential advantage that the number of behavioural states does not have to be specified a priori. Furthermore, assuming discrete behavioural states may impose lack of flexibility to the wide range of behaviours that animals can display.

Continuously varying movement states may also be achieved through time–warping [16], however our approach is arguably more interpretable as the parameters of the covariance kernel have clear biological meaning, while the hierarchical Gaussian process allows full propagation of uncertainty between layers. This approach may also be extended to include movement parameters that depend on environmental features as well as, or instead of, being driven by temporal patterns.

Fitting a model with smoothly varying parameters will be appropriate for situations where animals adopt a spectrum of different behaviours rather than switching between distinct, stereotyped behavioural modes. This would include movement behaviours that are driven by continuous variables such as temperature, vegetation quality, or water availability [61] that could either be included in the model as explicit covariates, or influence behaviour via seasonal or diurnal temporal patterns.

The computational complexity of Gaussian processes scales with the third power of the data set size. To deal with this issue, we have segmented the configuration space into local regions and have fitted a local GP to each. This is not just a simple heuristic, but improves the model flexibility in various respects, as previously discussed in the Statistics literature (see e.g. [62] and Chapter 9 in [63]).Various alternative and complementary methods have been proposed in the literature for dealing with the cubic scaling issue. For example, large classes of Matérn covariance functions can be approximated to arbitrary precision via Gaussian Markov random fields, which have sparse precision matrices [64]. Tapering and multi-resolution approximations are two additional approaches, developed for using Gaussian processes to analyze large spatial datasets [65, 66]. Gaussian processes may also be explicitly defined to have compactly-supported covariance functions, as in [67]. This has the effect of introducing zeroes into the covariance matrix, so that it can be efficiently manipulated using sparse matrix algorithms, thereby subtantially reducing the computational costs.

The present study has focused on non-parametric Bayesian modelling with Gaussian processes. Alternative parametric models have also been implemented, with splines, as applied in [27], particularly popular. The relation between splines and Gaussian processes has been discussed in the literature before; see e.g. [11], Section 6.3 and [58], Section 15.4. In essence, a splines based model is equivalent to the maximum a posterior (MAP) estimate of a Gaussian process whose covariance matrix is implicitly defined by the spline function and the spline regularizer. This has two disadvantages. The covariance matrix and hence the prior distribution in function space is not explicitly under the modeller’s control. In fact, [58], Section 15.4 provides examples of covariances matrices induced by splines that are rather unnatural and unsmooth. Moreover, the MAP estimate is a suboptimal substitute for full Bayesian inference, which in particular does not capture posterior estimation and prediction uncertainty. A practical advantage of splines over GPs is the reduction in the computational complexity, which is linear rather than cubic in the data set size. However, this advantage will become less relevant when reducing the GP computational complexity with the methods we employ or through the use of sparse GPs [68], whose application to animal movement modelling is a promising avenue for future work.

## Conclusions

The non-parametric nature of Gaussian process regression and the ability to model complex patterns through covariance kernels that can be combined through addition and multiplication [35], means arbitrary model structures can be learned. For example, if learning a migration route modelled as the mean of an Ornstein-Uhlenbeck process movement model, as in [69], our approach allows us to infer the mean location of the migration without restricting it to a particular functional form.

By specifying different covariance kernels we can introduce multiscale processes and periodicities into the model, such as diurnal or annual patterns, and combinations thereof.

The key advantages of probabilistic modelling with Gaussian processes are their equivalence to continuous time movement models, their non-parametric nature, and the availability of GPU-accelerated libraries for their implementation. Here we have demonstrated a multilayer Gaussian process approach to animal movement modelling that offers powerful and flexible inference of latent movement parameters for large scale data sets.

## Appendix

### Synthetic data generation

For simulations used in Sections 3.1 and 3.2, we simulate animal movement as a 2-dimensional Ornstein-Uhlenbeck process using the equation,

15 $$ d \mathbf{x}_{t} = -\nu_{t} \left(\mathbf{x}_{t} - \boldsymbol{m}_{t} \right) + \sigma_{t} d \mathbf{W}_{t}  $$

where **W**_*t*_ is a Weiner process and the parameters *ν*_*t*_,*σ*_*t*_ and ***m***_*t*_ are time varying properties of the stochastic process that correspond to the mean reversion rate, the amplitude of the noise term, and the mean location respectively. When simulating trajectories, we allow the parameters of the Ornstein Uhlenbeck process to vary in the following ways.

To model a diurnal periodic pattern of increased roaming at certain points of the day (Section 3.1), the noise amplitude *σ*_*t*_ is varied while all other parameters are held fixed with *ν*_*t*_=12 and the mean at the origin. The noise amplitude is created by modifying a sawtooth wave function with a 1-day period so that it increases linearly before dropping sharply at around 5pm. The wave function is then passed through a Gaussian smoother to obtain a more realistic pattern of activity.

To simulate an annual North-South migration (Section 3.2) we allow the *y*-component of mean location ***μ***_*t*_ to periodically oscillate between high and low latitudes for a single random walker. First, we create a smoothly oscillating function,

16 $$ g(t) = 1 + \cos{(2\pi t)}\sqrt{\frac{1+b^{2}}{1+b^{2}\cos^{2}({2\pi t})}}  $$

where *b* is a parameter that controls the smoothness of the transition between end-points of the migration (we set *b*=5) and *t* is measured in years. Next, we apply a slowly varying factor to the migration to model a shifting route,

17 $$ m_{t}^{y} = \left(5 - \exp{\left(-t/3\right)}\right) g(t)  $$

with $m_{t}^{x}=0$. The result is a periodic seasonal migration with a Northern area that is gradually shifting further North over several years. Figure S1 illustrates the resulting mean location of the migration route and an example trajectory for a single year.

### Empirical data

As part of an ongoing research project aimed at linking behaviour and space use with individual fitness, an entire flock of 60 merino sheep are being monitored in a large enclosure of 700 hectares near Pilcaniyeu in Northern Patagonia. Sheep have been equipped with GPS devices attached to collars (CatLog-B, Perthold Engineering, www.perthold.de; USA) and programmed to acquire locations every 10 min. 27 individuals with 2 months of data were selected for analysis. Data was collected between 20th May 2018 and 19th July 2018.

### Simulation of inferred models for empirical data

Using the inferred latent lengthscales and amplitudes from the sheep data, we simulated an equivalent Ornstein-Uhlenbeck process over the course of 20,000 days, with each day’s latent time-varying movement parameters being a different draw from the posterior distribution over the functions from the MCMC sampler.

The algorithm therefore involved making a single draw from the posterior distribution of both the lengthscale function and the amplitude function for each simulated day. These sample functions were converted to the movement parameters of the OU process by sampling the functions at discrete timepoints corresponding to the timesteps of the simulation. The velocity was then modelling as an OU process that was updated at each timestep using the parameter values for that timestep. Velocities were then integrated to form a trajectory that was down sampled to match the GPS collar schedule. We then compared summary statistics from the simulations to the raw data.

We looked at how well the model predicted the positional data from the GPS collars. We took the simulated trajectories and divided them into a sequence of discrete steps of 2 and 4 hours duration. We then compared the distribution of step lengths and turn angles between simulated and empirical data. Results are shown in Fig. S6. This comparison shows that there is good agreement between the distributions however the model underestimates the tendency for individuals to move in diametrically opposite headings. This is likely due to the effects of environmental features and site fidelity in the empirical data that the model is unable to capture.

## Supplementary Information

**Additional file 1** **Figure S1**: The simulated seasonal migration. (A) The mean location of the migration as a function of time. Note, there is a seasonal to-and-fro migration with a gradually shifting Northern range. (B) An example trajectory for a single year. The outbound and inbound movements of a single individual are shown in red and blue respectively. **Figure S2**: Convergence diagnostics for sampler. Potential scale reduction factor for 4 separate runs of 500 samples. (A-B) Raw whitened variables for the lengthscale (red) and amplitude (blue) (C-D) Transformed variables after Cholesky transformation for lengthscale (red) and amplitude (blue). Potential scale reduction factor for the observation noise was computed as 1.005. **Figure S3**: Effective sample size for 2000 MCMC samples from 4 chains. **Figure S4**: Posterior distribution of observation error related to the GPS locations. **Figure S5**: Posterior mean and 95% credible intervals of raw latent functions (prior to exponential transformation). Low value regions have high uncertainty as any movements in the trajectory in these periods are attributed to observation error. (A) Log correlation length. (B) Log velocity amplitude. **Figure S6**: Step lengths and turn angles from simulated and empirical data. Data was divided into sequences of 2-hour sections (A-B) and 4-hour sections (C-D). The distribution of lengths of segments and turn angles between segments is shown.

## Data Availability

Research Data (http://dx.doi.org/10.5525/gla.researchdata.1115). Source code is available here http://github.com/ctorney/moveNSGP
