# Supplementary figures and images for: A hierarchical machine learning framework for the analysis of large scale animal movement data
Source: Mov Ecol. 2021 Feb 18;9:6. doi: 10.1186/s40462-021-00242-0 (PMC7893961; doi:10.1186/s40462-021-00242-0)

A

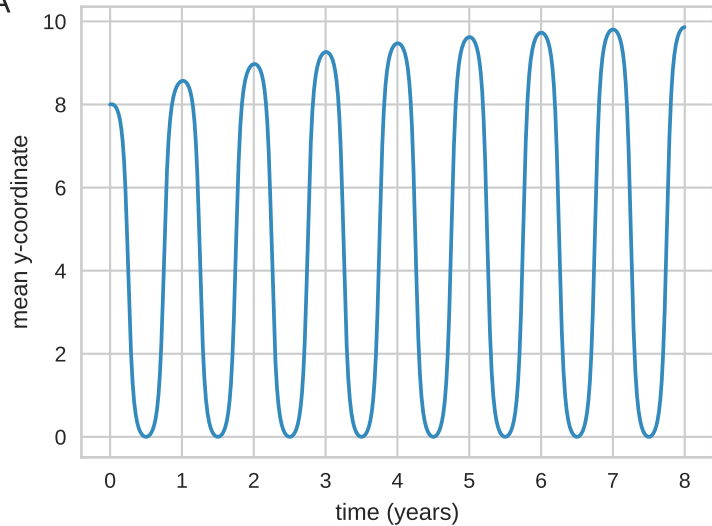

B

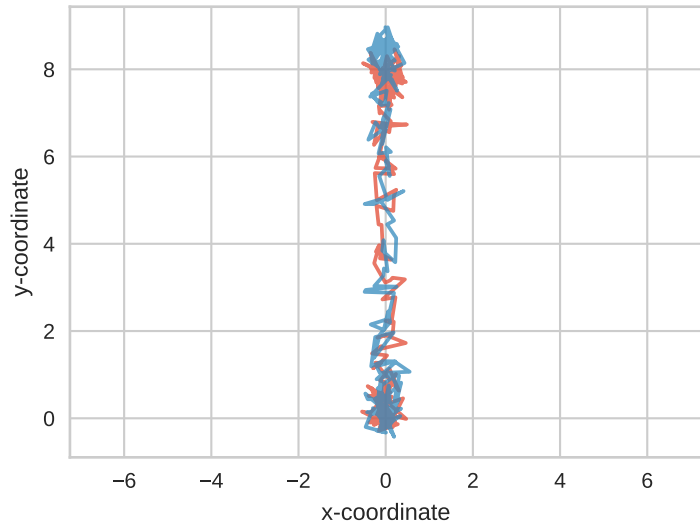

Supplement: Supplementary file 1 — Additional file 1 Figure S1: The simulated seasonal migration. (A) The mean location of the migration as a function of time. Note, there is a seasonal to-and-fro migration with a gradually shifting Northern range. (B) An example trajectory for a single year. The outbound and inbound movements of a single individual are shown in red and blue respectively. Figure S2: Convergence diagnostics for sampler. Potential scale reduction factor for 4 separate runs of 500 samples. (A-B) Raw whitened variables for the lengthscale (red) and amplitude (blue) (C-D) Transformed variables after Cholesky transformation for lengthscale (red) and amplitude (blue). Potential scale reduction factor for the observation noise was computed as 1.005. Figure S3: Effective sample size for 2000 MCMC samples from 4 chains. Figure S4: Posterior distribution of observation error related to the GPS locations. Figure S5: Posterior mean and 95% credible intervals of raw latent functions (prior to exponential transformation). Low value regions have high uncertainty as any movements in the trajectory in these periods are attributed to observation error. (A) Log correlation length. (B) Log velocity amplitude. Figure S6: Step lengths and turn angles from simulated and empirical data. Data was divided into sequences of 2-hour sections (A-B) and 4-hour sections (C-D). The distribution of lengths of segments and turn angles between segments is shown. [file 40462_2021_242_MOESM1_ESM.zip › 40462_2021_242_MOESM1_ESM/figS1.pdf]

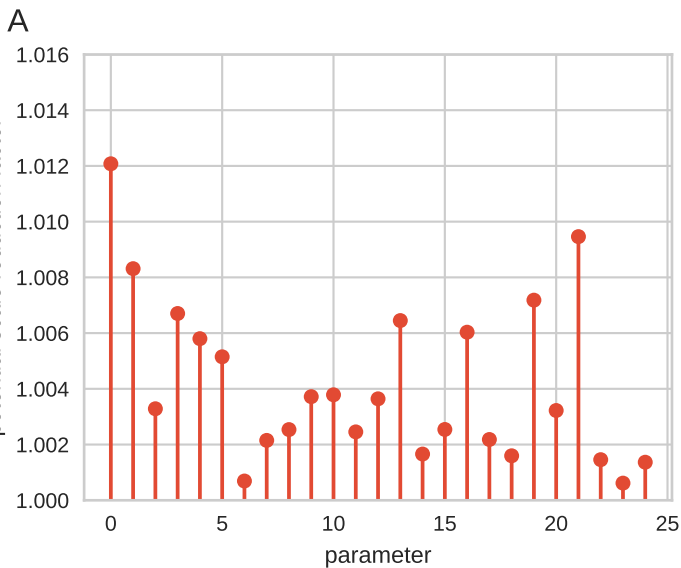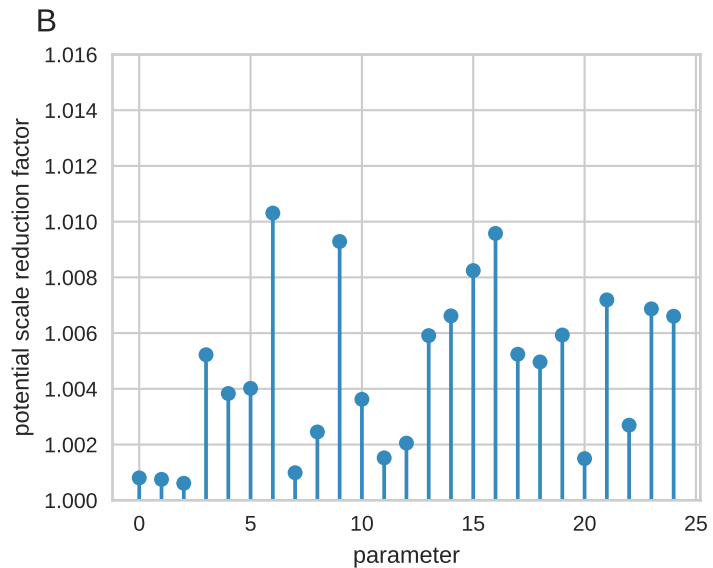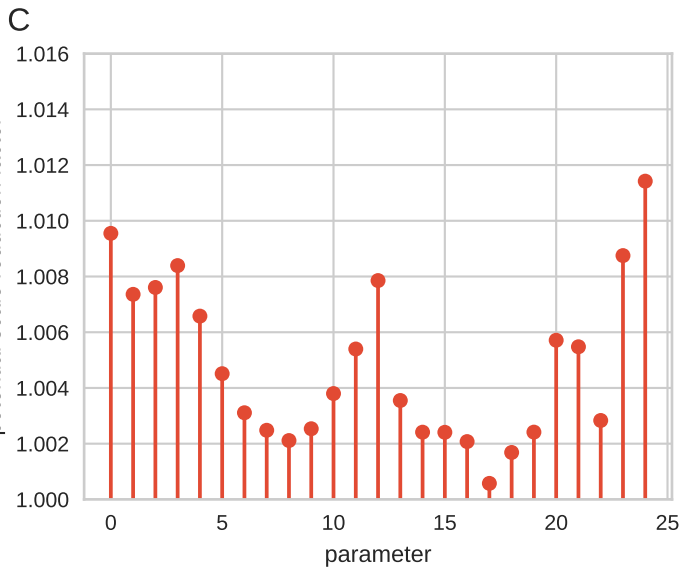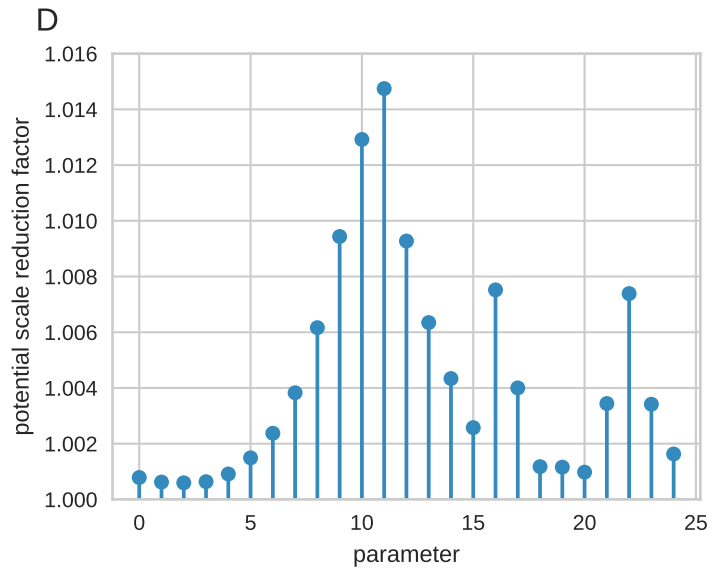

Supplement: Supplementary file 1 — Additional file 1 Figure S1: The simulated seasonal migration. (A) The mean location of the migration as a function of time. Note, there is a seasonal to-and-fro migration with a gradually shifting Northern range. (B) An example trajectory for a single year. The outbound and inbound movements of a single individual are shown in red and blue respectively. Figure S2: Convergence diagnostics for sampler. Potential scale reduction factor for 4 separate runs of 500 samples. (A-B) Raw whitened variables for the lengthscale (red) and amplitude (blue) (C-D) Transformed variables after Cholesky transformation for lengthscale (red) and amplitude (blue). Potential scale reduction factor for the observation noise was computed as 1.005. Figure S3: Effective sample size for 2000 MCMC samples from 4 chains. Figure S4: Posterior distribution of observation error related to the GPS locations. Figure S5: Posterior mean and 95% credible intervals of raw latent functions (prior to exponential transformation). Low value regions have high uncertainty as any movements in the trajectory in these periods are attributed to observation error. (A) Log correlation length. (B) Log velocity amplitude. Figure S6: Step lengths and turn angles from simulated and empirical data. Data was divided into sequences of 2-hour sections (A-B) and 4-hour sections (C-D). The distribution of lengths of segments and turn angles between segments is shown. [file 40462_2021_242_MOESM1_ESM.zip › 40462_2021_242_MOESM1_ESM/figS2.pdf]

A

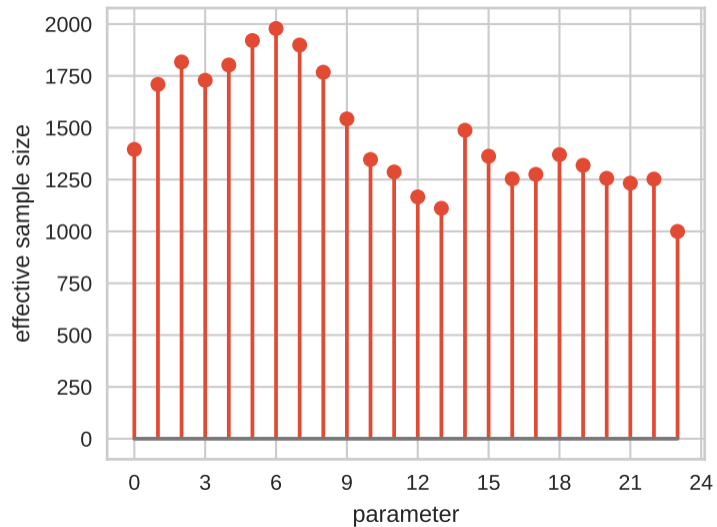

B

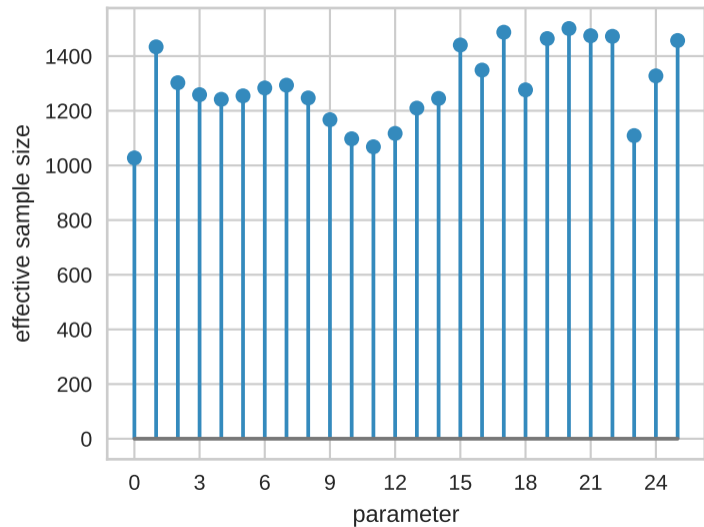

Supplement: Supplementary file 1 — Additional file 1 Figure S1: The simulated seasonal migration. (A) The mean location of the migration as a function of time. Note, there is a seasonal to-and-fro migration with a gradually shifting Northern range. (B) An example trajectory for a single year. The outbound and inbound movements of a single individual are shown in red and blue respectively. Figure S2: Convergence diagnostics for sampler. Potential scale reduction factor for 4 separate runs of 500 samples. (A-B) Raw whitened variables for the lengthscale (red) and amplitude (blue) (C-D) Transformed variables after Cholesky transformation for lengthscale (red) and amplitude (blue). Potential scale reduction factor for the observation noise was computed as 1.005. Figure S3: Effective sample size for 2000 MCMC samples from 4 chains. Figure S4: Posterior distribution of observation error related to the GPS locations. Figure S5: Posterior mean and 95% credible intervals of raw latent functions (prior to exponential transformation). Low value regions have high uncertainty as any movements in the trajectory in these periods are attributed to observation error. (A) Log correlation length. (B) Log velocity amplitude. Figure S6: Step lengths and turn angles from simulated and empirical data. Data was divided into sequences of 2-hour sections (A-B) and 4-hour sections (C-D). The distribution of lengths of segments and turn angles between segments is shown. [file 40462_2021_242_MOESM1_ESM.zip › 40462_2021_242_MOESM1_ESM/figS3.pdf]

posterior density

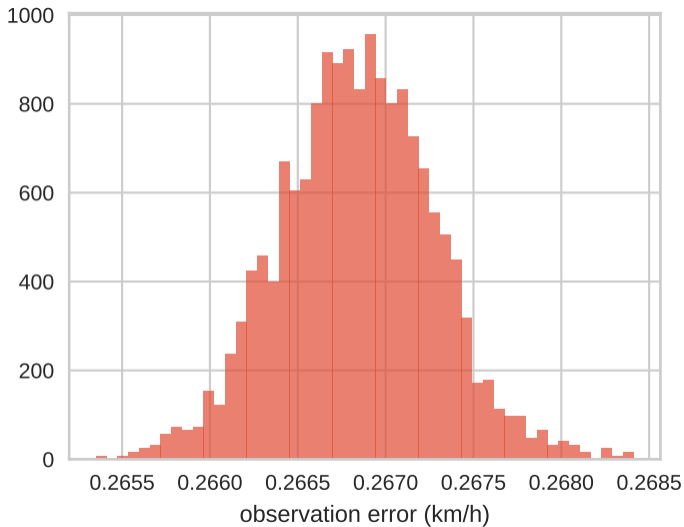

Supplement: Supplementary file 1 — Additional file 1 Figure S1: The simulated seasonal migration. (A) The mean location of the migration as a function of time. Note, there is a seasonal to-and-fro migration with a gradually shifting Northern range. (B) An example trajectory for a single year. The outbound and inbound movements of a single individual are shown in red and blue respectively. Figure S2: Convergence diagnostics for sampler. Potential scale reduction factor for 4 separate runs of 500 samples. (A-B) Raw whitened variables for the lengthscale (red) and amplitude (blue) (C-D) Transformed variables after Cholesky transformation for lengthscale (red) and amplitude (blue). Potential scale reduction factor for the observation noise was computed as 1.005. Figure S3: Effective sample size for 2000 MCMC samples from 4 chains. Figure S4: Posterior distribution of observation error related to the GPS locations. Figure S5: Posterior mean and 95% credible intervals of raw latent functions (prior to exponential transformation). Low value regions have high uncertainty as any movements in the trajectory in these periods are attributed to observation error. (A) Log correlation length. (B) Log velocity amplitude. Figure S6: Step lengths and turn angles from simulated and empirical data. Data was divided into sequences of 2-hour sections (A-B) and 4-hour sections (C-D). The distribution of lengths of segments and turn angles between segments is shown. [file 40462_2021_242_MOESM1_ESM.zip › 40462_2021_242_MOESM1_ESM/figS4.pdf]

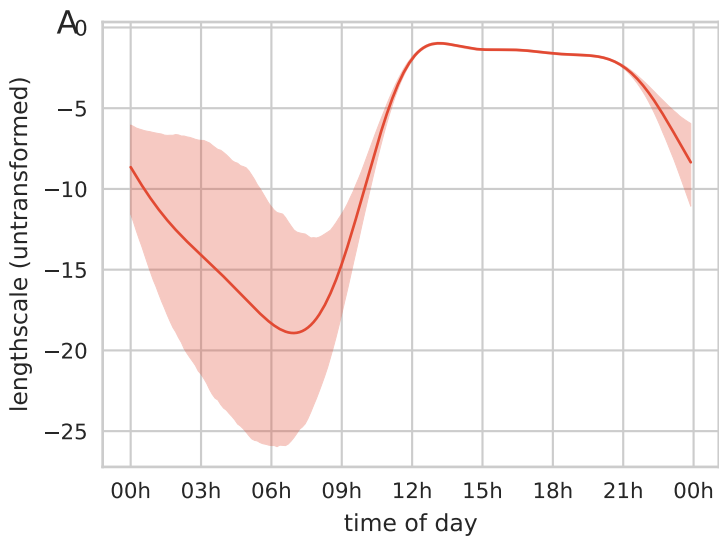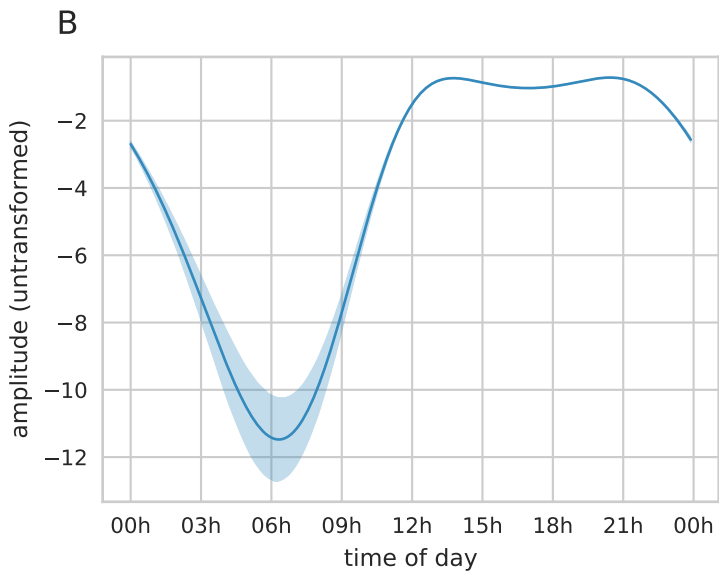

Supplement: Supplementary file 1 — Additional file 1 Figure S1: The simulated seasonal migration. (A) The mean location of the migration as a function of time. Note, there is a seasonal to-and-fro migration with a gradually shifting Northern range. (B) An example trajectory for a single year. The outbound and inbound movements of a single individual are shown in red and blue respectively. Figure S2: Convergence diagnostics for sampler. Potential scale reduction factor for 4 separate runs of 500 samples. (A-B) Raw whitened variables for the lengthscale (red) and amplitude (blue) (C-D) Transformed variables after Cholesky transformation for lengthscale (red) and amplitude (blue). Potential scale reduction factor for the observation noise was computed as 1.005. Figure S3: Effective sample size for 2000 MCMC samples from 4 chains. Figure S4: Posterior distribution of observation error related to the GPS locations. Figure S5: Posterior mean and 95% credible intervals of raw latent functions (prior to exponential transformation). Low value regions have high uncertainty as any movements in the trajectory in these periods are attributed to observation error. (A) Log correlation length. (B) Log velocity amplitude. Figure S6: Step lengths and turn angles from simulated and empirical data. Data was divided into sequences of 2-hour sections (A-B) and 4-hour sections (C-D). The distribution of lengths of segments and turn angles between segments is shown. [file 40462_2021_242_MOESM1_ESM.zip › 40462_2021_242_MOESM1_ESM/figS5.pdf]

A

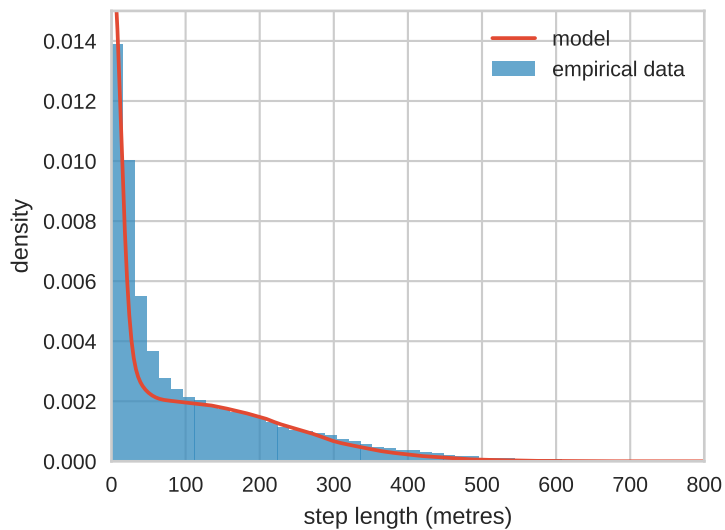

B

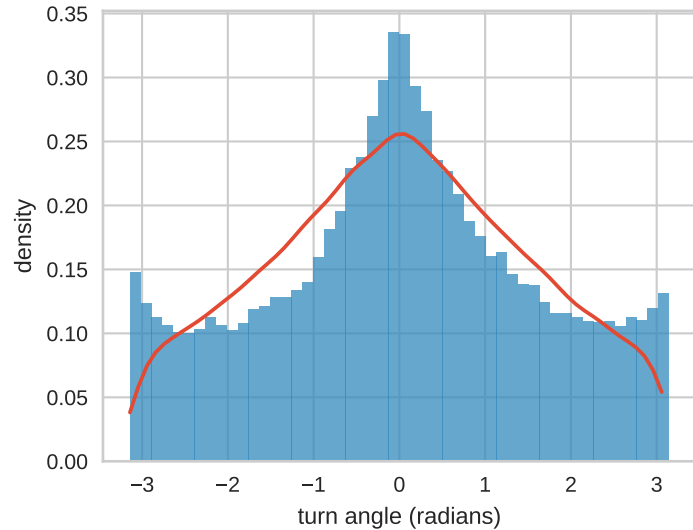

C

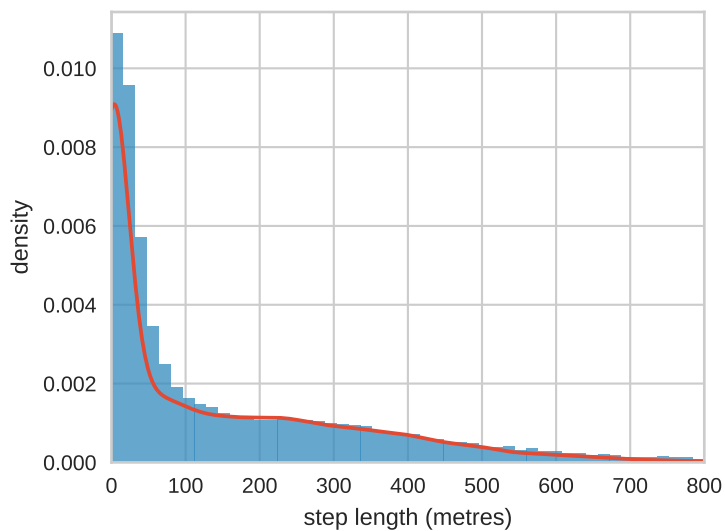

D

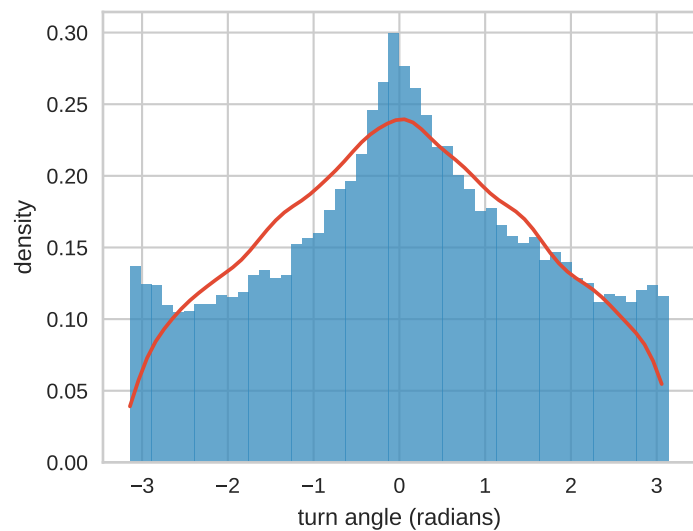

Supplement: Supplementary file 1 — Additional file 1 Figure S1: The simulated seasonal migration. (A) The mean location of the migration as a function of time. Note, there is a seasonal to-and-fro migration with a gradually shifting Northern range. (B) An example trajectory for a single year. The outbound and inbound movements of a single individual are shown in red and blue respectively. Figure S2: Convergence diagnostics for sampler. Potential scale reduction factor for 4 separate runs of 500 samples. (A-B) Raw whitened variables for the lengthscale (red) and amplitude (blue) (C-D) Transformed variables after Cholesky transformation for lengthscale (red) and amplitude (blue). Potential scale reduction factor for the observation noise was computed as 1.005. Figure S3: Effective sample size for 2000 MCMC samples from 4 chains. Figure S4: Posterior distribution of observation error related to the GPS locations. Figure S5: Posterior mean and 95% credible intervals of raw latent functions (prior to exponential transformation). Low value regions have high uncertainty as any movements in the trajectory in these periods are attributed to observation error. (A) Log correlation length. (B) Log velocity amplitude. Figure S6: Step lengths and turn angles from simulated and empirical data. Data was divided into sequences of 2-hour sections (A-B) and 4-hour sections (C-D). The distribution of lengths of segments and turn angles between segments is shown. [file 40462_2021_242_MOESM1_ESM.zip › 40462_2021_242_MOESM1_ESM/figS6.pdf]
